# Relation between acquisition of lexical concept and joint attention in children with autism spectrum disorder without severe intellectual disability

**DOI:** 10.1371/journal.pone.0266953

**Published:** 2022-04-14

**Authors:** Masuhiko Sano, Tetsu Hirosawa, Mitsuru Kikuchi, Chiaki Hasegawa, Sanae Tanaka, Yuko Yoshimura

**Affiliations:** 1 Department of Psychiatry and Neurobiology, Graduate School of Medical Science, Kanazawa University, Kanazawa, Japan; 2 Research Center for Child Mental Development, Kanazawa University, Kanazawa, Japan; 3 Faculty of Education, Institute of Human and Social Sciences, Kanazawa University, Kanazawa, Japan; Chiba Daigaku, JAPAN

## Abstract

In children with autism spectrum disorder (ASD), impairment of joint attention and language function are observed frequently from early childhood. Earlier reports have described these two phenomena as mutually related. For this study, developing past research, the relation between joint attention and the ability of conceptual inference is examined in 113 Japanese children (67.9 months mean age, 75% male) with ASD. We calculated Pearson’s correlation coefficients between their Joint attention abnormality evaluated by ADOS-2 and “Riddle” subscale in K-ABC, then they are negatively correlated: *r* (104) = -.285. A larger abnormality of joint attention is associated with a lower ability of conceptual inference. New findings were obtained indicating that, in children of this age group with ASD, the degree of joint attention impairment is correlated negatively with conceptual inference ability, but not with expressive and receptive language abilities. Consideration of the mechanism of this relation is presented in this report.

## Introduction

Autism spectrum disorder (ASD) is a neurodevelopmental disorder with widely diverse symptoms, including impaired social interaction and communication, restricted and repetitive patterns of behavior, and restricted and fixated interests [1].

Reportedly, many people with ASD have a deficiency in joint attention from infancy [2–5]. Joint attention is described as the ability to coordinate visual attention with another person and then shift the gaze toward an object or event. Joint attention is expressed by behaviors such as pointing with one finger to show another individual something interesting, showing another individual things by bringing them, or holding them up to share [6]. In typically developing (TD) children, joint attention is known to emerge from about 6 months and to become stable by about 9 months [7]. Joint attention is distinguished from behavior by the simple requirements that it include affection toward others and that it be related closely to frontal lobe function [3]. Dawson and colleagues extracted behaviors related to joint attention such as pointing and showing scenes from a video of a birthday party of a 1-year-old child. They evaluated the numbers of these behaviors. A group of children later diagnosed with ASD showed fewer joint attention behaviors than the TD group showed. Similar results were obtained when the ASD group was limited to IQ of 75 or higher [8]. Children with ASD are known to show various patterns of delay in language development such as late utterance of first word, poor response to their own name, and late development of expressive and receptive language from infancy [9].

As described above, children with ASD show impairment of joint attention and language development in infancy. We hypothesize that a relation exists between the impairment of joint attention and language development. Some earlier reports describe this relation. For example, Mundy and colleagues suggest that impairment of joint attention in early stages of development might be related to language developmental disorders that are specific to children with autism [2]. The study conducted by Mundy et al. investigated joint attention, non-joint attention social behavior, requesting behavior, language performance, intelligence in children with ASD (mean age of 44.9 months), and examined correlation between them and language performance one year later. Results show that only joint attention was found to have significant positive correlation with language performance one year later. The others did not. In addition, Siller and Sigman reported that, in children with ASD, joint attention behavior in childhood significantly predicts language performance in adolescence [10]. A meta-analysis by Bottema-Beutel also indicated that joint attention in children with ASD is more closely associated with language and is a predictor of both receptive and expressive languages, compared to neurotypical children [11].

For children with ASD after age three, the relation between language and joint attention remains unclear. Furthermore, in earlier studies, the language performance to be evaluated is mainly that of receptive languages and expressive languages. Other language functions that integrate these have rarely been evaluated [11]. It is particularly interesting that an earlier study of children with ASD (aged 5–7) [12] uses a task “riddle” of Kaufman Assessment Battery for Children [13] that reflects acquisition of vocabulary concepts, which is particularly impaired compared to that of TD children.

This study of children with ASD who are older than three years old was conducted to evaluate the relation between the development of joint attention and language ability related to the acquisition of vocabulary concepts. We hypothesized that, for children with ASD who are older than three years old, joint attention development is positively related to vocabulary conceptual inference ability evaluated by K-ABC.

## Method

### Participants

From Kanazawa University and affiliated hospitals, we recruited 153 children with ASD (116 boys, 37 girls, age 36–98 months). The ASD diagnosis was made according to the Diagnostic and Statistical Manual of Mental Disorders, fourth edition (DSM-IV) using the Diagnostic Interview for Social and Communication Disorders (DISCO) [14] or the Autism Diagnostic Observation Schedule second edition (ADOS-2) [15]. We excluded data of 14 children from statistical analyses: they were unable to complete the psychometric evaluation.

For this study, we attempted to evaluate joint attention using ADOS-2: a semi-structured, standardized assessment method that includes several play-based activities designed to obtain information in the areas of communication, reciprocal social interactions, and restricted and repetitive behaviors associated with a diagnosis of ASD. The ADOS-2 encompasses five modules, each with its own schedule of activities for participants with particular developmental and language capabilities, ranging from those who are preverbal or have minimal language skills (Module T and Module 1) or not verbally fluent children (Module 2) to verbally fluent children (Module 3), or adolescent and adults (Module 4) [15]. For this study, 16 children were evaluated using Module 1, 116 children using Module 2, and 7 children using Module 3. To unify the evaluation scale of joint attention, we included only 116 children evaluated using Module 2 in statistical analysis. The others were excluded. Additionally, to unify the intellectual function of our sample, we excluded three children for whom the K-ABC Mental processing scale was less than 60. Finally, we analyzed 113 children with ASD (85 boys, 28 girls, aged 40–98 months). Table 1 presents their characteristics. Written informed consent was obtained from parents before participation by the children. The Ethics Committee of Kanazawa University Hospital approved the methods and procedures, all of which were conducted in accordance with the Declaration of Helsinki.

**Table 1:** Demographic characteristics of study participants.

*N*	113
Gender (%male)	75%
Age in months	67.9 (11.1)
Height (cm)	111 (6.95)
Weight (kg)	19.1 (3.65)
K-ABC	
Mental processing scale	93.4 (18.2)
Achievement scale	93.8 (17.2)
ADOS-2	
SA	8.8 (3.5)
RRB	1.8 (1.4)
Total	10.7 (4.1)
CS	5.4 (1.9)

Numbers are mean (standard deviation) or counts.

K-ABC, Kaufman Assessment Battery for Children; ADOS-2, Autism Diagnostic Observation Schedule-2; SA, Social Affect; RRB, Restricted and Repetitive Behaviors; Cs, Comparison score.

### Language assessment

For this study, to evaluate the participants’ language ability, we used subscales “Expressive Vocabulary,” “Riddle” in the Japanese version of K-ABC, and the Picture Vocabulary Test-Revised (PVT-R). “Expressive Vocabulary” reflects the expressive ability to speak the correct names of object illustrations. “Riddles” reflects language conceptual inference ability. The PVT-R used to assess language comprehension is similar to the Peabody Picture Vocabulary Test-Revised (PPVT-R). We used PVT-R as the index of receptive language.

In K-ABC, “Expressive Vocabulary” is a subscale for children aged 36–71 months. The study participants were aged 67.9 months at means, so only 21 children were evaluated using this scale. Considering the small sample size, we excluded expressive language from the main statistical analyses.

### Joint attention assessment

To guarantee evaluator quality, ADOS-2 was used as the Gold Standard for ASD diagnoses. It is widely regarded as an appropriate method for evaluating large samples. In ADOS-2 Module 2 items, Pointing (item A-6), Gesture (item A-7), Unusual Eye Contact (item B-1), Showing (item B-5), and Initiation of Joint Attention (item B-6) were identified using factor analysis as factors of joint attention [16].

### Statistical analysis

We used the total score of Pointing (item A-6), Gesture (item A-7), Unusual Eye Contact (item B-1), Showing (item B-5), and Initiation of Joint Attention (item B-6) as “JA sum” to evaluate our participants’ joint attention. We calculated the JA sum based on the ADOS manual. We used JA sum as the scale of joint attention for the study described herein. JA sum was treated as a continuous variable with values of 0–10.

For scales of language functions, we used the standardized score of Riddle in K-ABC as a scale of conceptual inference ability, and used the evaluation score of PVT-R as a scale of receptive language. Pearson’s correlation coefficients were calculated between JA sum and two language scales (Riddle in K-ABC, PVT-R) using Bonferroni-adjusted *p*-values (*p*-value*2).

In addition, as a supplementary analysis, we evaluated the correlation between JA sum and the standardized score of Expressive Vocabulary in K-ABC similarly.

For these analyses, results for which *p* < .05 were inferred as significant. All statistical analyses were conducted using software (Stata/MP 16.0; Stata Corp., College Station, TX, USA).

## Result

**Fig 1:**
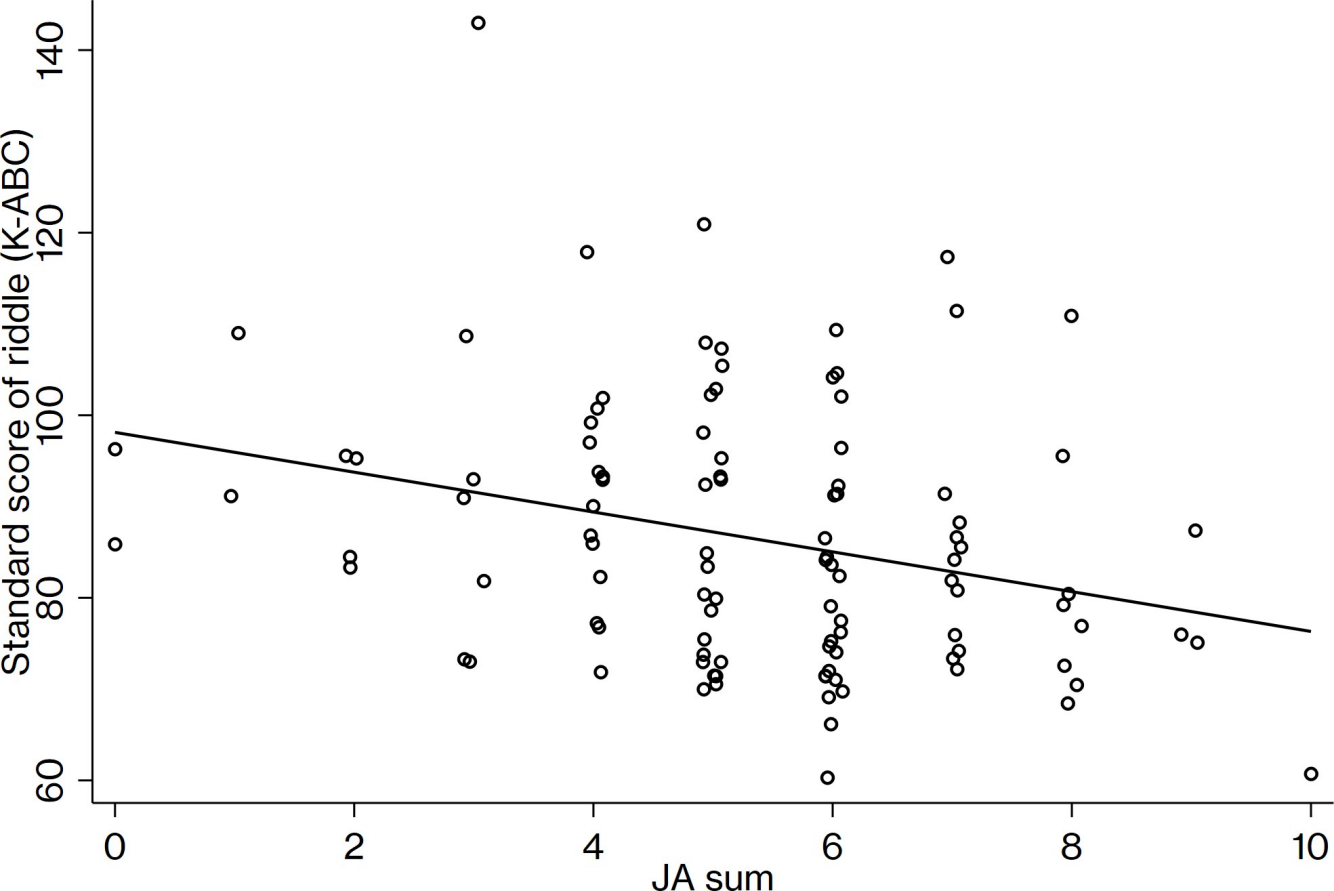
Relation between conceptual inference ability and Joint attention. Relation between conceptual inference (Riddle) on the Kaufman Assessment Battery for Children and ADOS-2 Joint Attention. JA sum, the total score of ADOS-2 Module 2 items, Pointing, Gesture, Unusual Eye contact, Showing, and Initiation of Joint Attention.

Results show JA sum and Riddle in K-ABC as negatively correlated: *r* (104) = -.285, adjusted *p*-value = .006. A larger abnormality of joint attention is associated with a lower ability of conceptual inference. No significant correlation was found between the JA sum and PVT-R: *r* (103) = -.096, adjusted *p*-value = .659. Fig 1 portrays a scatter plot and regression lines to illustrate the relation between joint attention and conceptual inference.

Supplementary analyses showed that Expressive Vocabulary in K-ABC was not significantly correlated to JA sum: *r* (19) = -.142, adjusted *p*-value = 1.620.

## Discussion

We examined the relation between joint attention and language function as evaluated by K-ABC in children with ASD aged 40–98 months (mean 67.9 months). Joint attention was correlated significantly with Riddle, but not with receptive language (i.e., PVT-R). These results are consistent with our hypothesis.

In the Riddle subtest, children were required to retrieve a target word according to suggested words that were associated conceptually with the target word. This subtest evaluates the development of conceptual formation and conceptual inference as well as word retrieval ability. In other words, conceptual inference (Riddle) is regarded as a higher language ability integrating expressive and receptive languages that is also related to knowledge gained through education and experience. Joint attention might affect this ability in children with ASD who are older than three years old after they acquire numerous vocabularies. We next consider the background of this relation.

First, the usage-based model suggests one hypothesis [17]. According to the usage-based model, human beings acquire symbols of words through accumulation of experience. They further acquire language by networks categorizing multiple symbols based on similarity and commonality. This dynamic network category has the property of constantly changing with experience even after it is formed. Based on Tomasello, children’s language acquisition arises from social interactions with adults. It relies on skills such as joint attention and intent understanding. Although children with ASD have communication deficiencies, those with severe joint attention impairment have less communication and experience sharing with others. Therefore, a unique network is formed and maintained. Consequently, their conceptual inference task scores on intelligence tests are low. In other words, deficiency of joint attention in children with ASD does not impair the ability of conceptual inference. Rather, it affects the formation of network categories different from those in TD children. If therapeutic interventions that improve joint attention deficiency children with ASD can be performed, then their conceptual inference is expected to be similar to that of TD children. Their function of linguistic communication would be improved.

Another hypothesis is that impairment of long-distance networks in the brain, which is frequently reported in children with ASD, affects both. Joint attention requires soundness of the network of the whole brain; conceptual inference also requires it. In the conceptual inference task, a participant is required to search for conceptually related words from the presented words and to answer them. The correct answer is sought by activating the network structure and by deriving the network of concepts accumulated as long-term memory. For example, a brain region might be involved simultaneously in both joint attention and conceptual analogy brain networks. Disorders in this region might affect both phenotypes simultaneously. Delbruck et al. compared the strength of brain network connectivity related to joint attention in individuals with ASD and TD using resting state functional MRI (fMRI). They reported that individuals with ASD showed weaker functional connectivity between regions such as the right lateral occipital temporal lobe and the right occipital lobe, the left lateral occipital temporal gyrus and the right fusiform gyrus, the left middle temporal gyrus and the right fusiform gyrus, and the right middle temporal gyrus and the right lateral occipital temporal gyrus [18]. Goelman investigated the brain network pathways activated during the joint attention task in healthy women of mean age of 24 using fMRI. Results clarified that the dorsomedial prefrontal cortex, ventromedial prefrontal cortex, temporoparietal junction, superior temporal sulcus, and posterior cingulate gyrus are related to this pathway [19]. Zhang et al. used fMRI in healthy individuals with mean age of 24 years to investigate brain regions that are activated during listening to a radio program. They found that different brain regions are activated for each semantic category of listening to words [20]. Furthermore, in their study, activation was observed in widely diverse brain regions that were found to be associated with joint attention in studies by Delbruck et al. and by Goelman et al., such as the medial frontal gyrus, posterior cingulate gyrus, middle temporal gyrus, and fusiform gyrus. Because of differences in the ages of participants and diagnosis in each study, combining these results simply to identify brain regions involved in both joint attention and conceptual inference is not possible. Further imaging studies must be conducted to investigate the possible common neural basis of joint attention and conceptual inference in ASD.

This study found no correlation between receptive language with joint attention. This result is inconsistent with results obtained from earlier studies showing correlation between joint attention in receptive language [2,10,11]. Possible causes are the age and intellectual level of the participants. Compared to earlier studies, this study targeted a slightly older age group without severe intellectual disability. In such a group, even in children with severe joint attention impairment, receptive language might be compensated. Another possible cause is the test battery. For this study, joint attention was evaluated using ADOS. Language function was evaluated using K-ABC and PVT-R. This combination has not been used in earlier studies that include evaluation of relations between joint attention and receptive language. This combination might be insufficient to evaluate this relation.

This study has some limitations. First, the relation between joint attention and expressive language ability could not be evaluated because of the small sample size. As such, it remains unclear whether the relation between joint attention and conceptual inference ability was affected by expressive language ability. Next, we did not evaluate comorbid disorders such as attention-deficit hyperactivity disorder, movement disorder, and coordination disorder, which can affect joint attention. For instance, comorbid ADHD was reported as augmenting ASD symptoms [21]. Also, ADHD children showed different patterns of eye movements compared to TD children [22]. These comorbid disorders are considered to be important factors for controlling gaze and social interest. Therefore, ADHD and movement disorder evaluation must be involved in future studies. Finally, this study demonstrated only the correlation between joint attention behavior and conceptual inference ability in a test battery, not the causal relation by which joint attention development positively affects lexical concept acquisition. In other words, it remains unknown whether interventions that promote joint attention change the ability of conceptual inference. Future studies must elucidate whether interventions promote joint attention and facilitate linguistic competence using early intervention techniques such as the Early Start Denver Model [23] and Joint Attention, Symbolic Play, Engagement and Regulation [24], which were designed to promote early social development, including joint attention. These emphasize typical developmental processes, not only to look at, but for development of interpersonal relationships. In future studies, we should investigate whether children who have taken such an approach to support early interpersonal relationship development will be promoted significantly not only in social adaptation but also in the acquisition of lexical concepts. If such intervention were found to improve verbal communication, which is a central impairment of ASD, it would be hopeful news for all involved in ASD.

## Supporting information

S1 File (XLS)
